# The role of FGF-2 and BMP-2 in regulation of gene induction, cell proliferation and mineralization

**DOI:** 10.1186/1749-799X-6-8

**Published:** 2011-02-09

**Authors:** Millie Hughes-Fulford, Chai-Fei Li

**Affiliations:** 1 Department of Research, Veterans Affairs Medical Center, 4150 Clement Street, San Francisco, CA 94121, USA; 2 Department of Medicine, University of California, 4150 Clement Street, San Francisco,, CA 94121, USA; 3 Department of Urology, University of California, 4150 Clement Street, San Francisco,, CA 94121, USA; 4 Hughes-Fulford Laboratory, Northern California Institute for Research and Education, 4150 Clement Street, San Francisco, CA 94121, USA

## Abstract

**Introduction:**

The difficulty in re-growing and mineralizing new bone after severe fracture can result in loss of ambulation or limb. Here we describe the sequential roles of FGF-2 in inducing gene expression, cell growth and BMP-2 in gene expression and mineralization of bone.

**Materials and methods:**

The regulation of gene expression was determined using real-time RTPCR (qRTPCR) and cell proliferation was measured by thymidine incorporation or fluorescent analysis of DNA content in MC3T3E1 osteoblast-like cells. Photomicroscopy was used to identify newly mineralized tissue and fluorescence was used to quantify mineralization.

**Results:**

Fibroblast growth factor-2 (FGF-2) had the greatest ability to induce proliferation after 24 hours of treatment when compared to transforming growth factor beta (TGFβ, insulin-like growth factor-1 (IGF-1), bone morphogenic protein (BMP-2), platelet derived growth factor (PDGF) or prostaglandin E_2 _(PGE_2_). We found that FGF-2 caused the most significant induction of expression of early growth response-1 (*egr-1), fgf-2, cyclo-oxygenase-2 (cox-2), tgfβ and *matrix metalloproteinase-3 *(mmp-3) *associated with proliferation and expression of angiogenic genes like vascular endothelial growth factor A (*vegfA) *and its receptor *vegfr1*. We found that FGF-2 significantly reduced gene expression associated with mineralization, e.g. *collagen type-1 (col1a1), fibronectin (fn), osteocalcin (oc), IGF-1, noggin, bone morphogenic protein (bmp-2) and alkaline phosphatase (alp)*. In contrast, BMP-2 significantly stimulated expression of the mineralization associated genes but had little or no effect on gene expression associated with growth.

**Conclusions:**

The ability of FGF-2 to re-program a mineralizing gene expression profile to one of proliferation suggests that FGF-2 plays a critical role of osteoblast growth in early fracture repair while BMP-2 is instrumental in stimulating mineralization.

## Introduction

The mechanisms that regulate bone growth and mineralization remain poorly understood. The cellular events involved in bone formation include chemotaxis of osteoblast precursors, growth factor (GF) production, proliferation of committed osteoblast precursors, and the differentiation (mineralization) of osteoblasts. Bone formation requires expression of structural proteins such as collagen type I, osteocalcin, noggin and runx2 during mineralization [1]. Numerous studies suggest that a variety of growth factors such as FGF-2, TGFβ, IGF-1, PDGF and PGE_2 _act as autocrine and paracrine hormones to regulate bone cell proliferation [2]. FGF-2 is an important modulator of bone formation *in vitro *and *in vivo *[3,4]. FGF-2 is tightly bound to the bone matrix and can be extracted as a biologically active GF [5] and is thought to play a major role in wound healing [6,7].

To evaluate the physiological activity of FGF-2 and other growth factors, we studied their relative ability to influence proliferation of osteoblasts at a site of injury in a mineralized culture. MC3T3-E1 is a cloned mouse osteoblast-like cell line that retains synthetic functions of bone. When treated with differentiation media, these cultured osteoblasts have the ability to differentiate, including synthesis of alkaline phosphatase [8], type I collagen [9], osteocalcin [10,11] and mineralized matrix containing hydroxyapatite crystals [12].

We have previously reported that FGF-2 is induced by mechanical stress [13,14] and causes proliferation after mechanical stress. FGF-2 is an immediate-early gene that is regulated by both PKA and MAPK signal transduction pathways [15]. Here we report that FGF-2 induces expression of growth-related genes and down-regulates genes responsible for differentiation and mineralization. In addition, BMP-2 is considerably more effective than FGF-2 in inducing new mineralization.

## Materials and methods

### Materials

We obtained GFs from Amgen, Thousand Oaks, CA. FGF-2 and IGF-1 from R & D Systems, Minneapolis, MN. TGFβ, PDGF and dmPGE_2 _are from Cayman Chemical, Ann Arbor, Michigan. Cell culture supplies (αMEM, fetal calf serum, trypsin and antibiotics) were obtained through the tissue culture facility at the University of California, San Francisco. Cell culture dishes were purchased from Corning, Corning, New York. Rhodamine-phalloidin is from Invitrogen, Carlsbad, California. Tritiated thymidine and 35 S methionine are from Amersham, Arlington Heights, IL. All other materials came from standard laboratory suppliers. MC3T3E1 osteoblast-like cells, a cloned cell line, established by Kodama [8,12] were used in this study at early passage number.

### Methods

We maintained cloned MC3T3-E1 osteoblast-like cells in normal media (NM) consisting of alpha MEM medium with 10% fetal calf serum (FCS), 1% antibiotic solution and 1% glutamine solution and subcultured the cells every 3 to 4 days. The cells were subcultured by incubating with trypsin for five minutes and resuspending at a concentration of 3 × 105 cells/ml. For experiments, we grew the cells in the NM above, using multi-well plates. After three days, the cells reach confluence and mineralization medium (MM) was added. MM is alpha MEM medium with 5% fetal calf serum (FCS), 1% antibiotic solution and 1% glutamine solution supplemented with ascorbic acid (50 μg/ml) and β-glycerol phosphate (10 mM) to support mineralization. The cultures were then incubated for 1-2 more days for mineralization studies. We used at least triplicate independent biological samples in multiple experiments for data collection.

### Protein Assay

Protein concentration was determined by Bio-Rad DC protein assay (Bio-Rad, CA) according to manufacturer's protocol.

### Microscopy

At the conclusion of the 24 or 48 hour incubation, the coverslip was removed. The specimen was rinsed five times in room temperature phosphate buffered saline (PBS) and fixed. We then visualized the mineralizing cells with 2% Alizarin Red. After rinsing in distilled water and air drying the samples, we mounted the coverslips on microscope slides using Fluoromount and examined and photographed the cells on a Zeiss Axioskop using 20×.

### Tritiated thymidine incorporation into DNA

At the conclusion of the 24 hour incubation, the culture medium was removed and the cells were incubated for 15 minutes at 37°C in 1 ml PBS containing tritiated thymidine (4 μCi/ml) as described previously [16]. Following this incubation, the PBS was removed and the cells were washed 3 times with ice cold trichloroacetic acid (TCA) followed by ice cold ethanol and allowed to air dry. Then 1 ml of sarkosyl lysing buffer was added to each well; all the cells were solubilized after 30 minutes. Finally, after mixing the resulting solution with a pipette, radioactivity was counted in a scintillation counter and protein content was measured. The data was calculated and expressed as disintegrations per minute (DPM) per microgram protein.

### Alizarin Red visualization of mineralization

Alizarin Red (2%) stained cells were incubated with 10% acetic acid for 30 minutes to release bound Alizarin Red into solution. The solution was neutralized with 10% ammonium hydroxide and the absorbance of Alizarin Red was measured at 450 nm using a microplate reader. Data is expressed in absolute amounts according to a standard curve.

### RNA Isolation

RNA were isolated through the use of the RNeasy™Mini kit (QIAGEN, Valencia, CA) or TriReagent™ according to the manufacturer's protocol. For RNeasy™ Mini kit RNA isolation, cells were seeded in 6-well plates with αMEM media supplemented with 10% FCS, then downregulated and activated as indicated in the figure legends. Cells were lysed using 350 μl of buffer RLT (supplied in kit) containing 2-mercaptoethanol (Biorad, Hercules, CA). The lysate was then placed into QIAshredder homogenizer (QIAGEN, Valencia, CA) and centrifuged at 20,000 rpm for 2 minutes. 350 μl of 70% ethanol was added to the flow through, mixed, and centrifuged in the RNeasy™Mini column (supplied in kit) for 15 s at 20,000 rpm. Flow through was discarded and the column was washed with 700 μl of buffer RW1 (supplied in kit) for 15 s at 20,000 rpm. Two additional washes were performed with 500 μl of buffer RPE (supplied in kit) at 20,000 rpm for 15 s and 2 minutes, respectively. The flow through was discarded and the column placed in a sterile 1.5 ml collection tube. Depending on the expected yield, 20-50 μl RNase-free water is pipetted into the column and centrifuged for 1 minute at 20,000 rpm. The samples are then stored at -80°C until further analysis.

### Reverse Transcription (RT)

1.5 μg of RNA was added to 30 μl reverse transcriptase (RT) reaction buffer containing 5 mM MgCl_2_, 10 mM Tris-HCl (pH 8.3), 50 mM KCl, 1 mM dNTPs, 2.5 μM oligo d(T) primer, 2.5 U/μl of MuLV, and 1 U/μl of RNase inhibitor. The RT reaction was incubated at room temperature for 10 min, 42°C for 30 min, inactivated at 99°C for 5 min, and cooled at 5°C for 5 min.

### Real-time Quantitative RT-PCR Reaction (qRTPCR)

2 μl of cDNA from the RT reaction was added to 20 μl real-time quantitative polymerase chain reaction (qPCR) mixture containing 10 μl of 2× SYBR^® ^Green PCR Master Mix (Applied Biosystems, Foster City, CA) and 12 pmol oligonucleotide primers. PCRs were carried out in a Bio-Rad MyiQ Single-Color Real-Time PCR Detection System (Bio-Rad, Hercules, CA). The thermal profile was 50°C for 2 min, 95°C for 10 min to activate the Taq polymerase, followed by 50 amplification cycles, consisting of denaturation at 95°C for 1 min 40 s, annealing at 63°C for 1 min 10 s and elongation at 72°C for 1 min 40 s. Fluorescence was measured and used for quantitative purposes. At the end of the amplification period, melting curve analysis was performed to confirm the specificity of the amplicon. RNA samples were normalized to *cyclophilin (CPHI) *internal standard. Relative quantification of gene expression was calculated by using 2^-(Ct gene T - Ct CPHI T)-(Ct gene 0 hr - Ct CPHI 0 hr) ^equation, where "C_t _gene T" represents the calculated threshold cycle (C_t_) of a time point of each sample other than 0 hr, or each treatment other than control. Relative gene absolute abundance was calculated using 2^(Ct gene T - Ct CPHI T) ^as previously described [17] allows us to compare the abundance of the gene between other genes and experiments. The resulting numbers were then multiplied by 10,000 for better graphical presentation. Primer sequence information was previously published [18-22]. All data derived using qRTPCR was from multiple experiments with at least triplicate independent biological samples.

## Results

### Growth factor effect on cell proliferation DNA synthesis

As seen in Table 1, in the absence of any added compounds there were small and unremarkable changes in DNA synthesis with IGF-1 and PDGF; in contrast, FGF-2, TGFβ and PGE2 significantly enhanced thymidine incorporation within 24 hours of treatment. TGFβ stimulated thymidine incorporation more than 2 fold while FGF-2 and PGE2 increased DNA synthesis more than 4.5 and 3.3 fold respectively.

**Table 1:** Effect of growth factors on protein synthesis in wounded mineralized osteoblasts.

Treatment	Thymidine incorporation DPM × 10^3^/ug protein
con	37.6 ±2.9
IGF-1	42.3 ± 4.2
FGF-2	114.3 ± 11
TGFβ	65.2 ± 12
PDGF	39.8 ± 7.2
BMP-2	41.5 ± 5.6
PGE2	84.1 ± 23.1

Representative experiment showing the effects of IGF-1 (20 ng/ml), FGF-2 (2.0 ng/ml), TGFβ (2 ng/ml), PDGF (3 ng/ml), BMP-2 (100 ng), PGE_2 _(2 μg/ml) on proliferation/mg protein of MC3T3-E1 osteoblasts after 24 hours of treatment (n = 4).

### Regulation of FGF-2 induced gene expression

**Figure 1:**
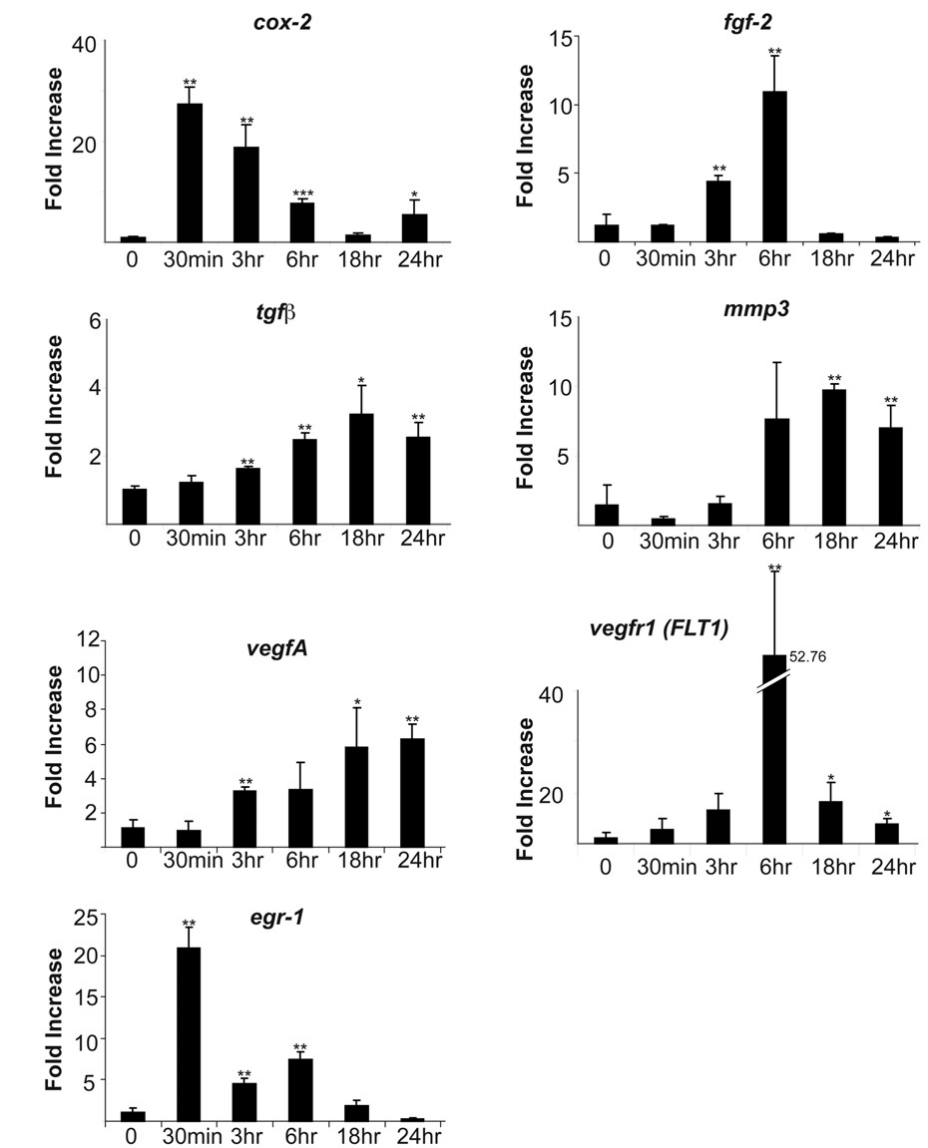
**qRTPCR analysis of gene induction of proliferation and angiogenesis**; qRTPCR analysis of gene reduction of genes over 24 hours of treatment with FGF-2 shows a significant increase in genes associated with proliferation and angiogenesis. Cultures were cultured and harvested for RNA as described in Materials and Methods. Each bar represents mean ± SD triplicate independent biological samples each time point corrected to cyclophilin. (*p < 0.05; **p < 0.01 with two-tail student t-test compared to 0 hour of each gene)

Using qRTPCR, we found that FGF-2 dramatically induced *egf-1*, *fgf-2*, *cox-2, tgfβ, mmp3, vegfA and vegfr1 *over a 24 hour period each displaying a different sequential temporal pattern of gene induction (Figure 1). *VegfA and vegfr1 *are associated with angiogenesis while *mmp3*, is associated with increased migration. *Tgfβ, fgf-2, egr-1 and cox-*2 are key genes in regulation of osteoblast proliferation.

**Figure 2:**
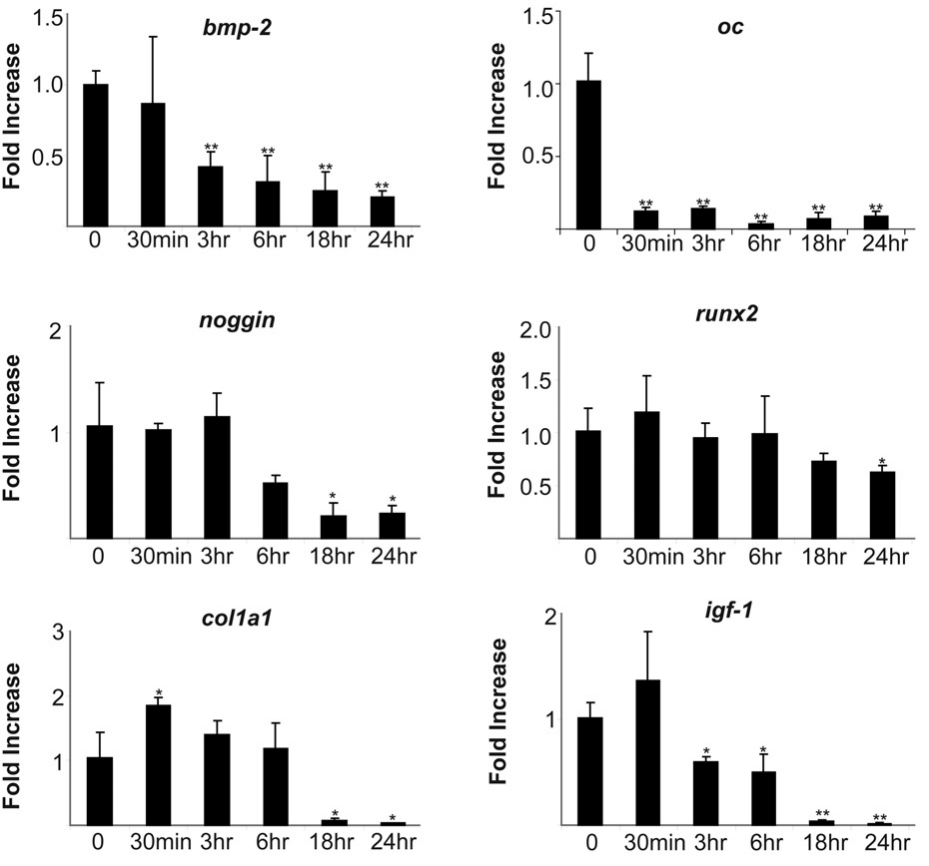
**qRTPCR analysis of FGF-2 regulated genes associated with mineralization**; qRTPCR analysis of gene reduction of genes over a 24 hours of treatment with FGF-2 shows a marked reduction in genes associated with mineralization. Cultures were cultured and harvested for RNA as described in Materials and Methods. Each bar represents mean ± SD triplicate independent biological samples at each time point corrected to cyclophilin. (*p < 0.05; **p < 0.01 with two-tail student t-test compare to 0 hour of each gene.)

Interestingly, we found that FGF-2 also significantly decreased expression of other genes associated with mineralization including *col1a1, fn, bmp-2, oc, run-x, and noggin*. IGF-1, a known differentiation factor, was significantly decreased by FGF-2 treatment. (Figure 2).

### Relative abundance of genes regulated by FGF-2 and BMP-2

**Figure 3:**
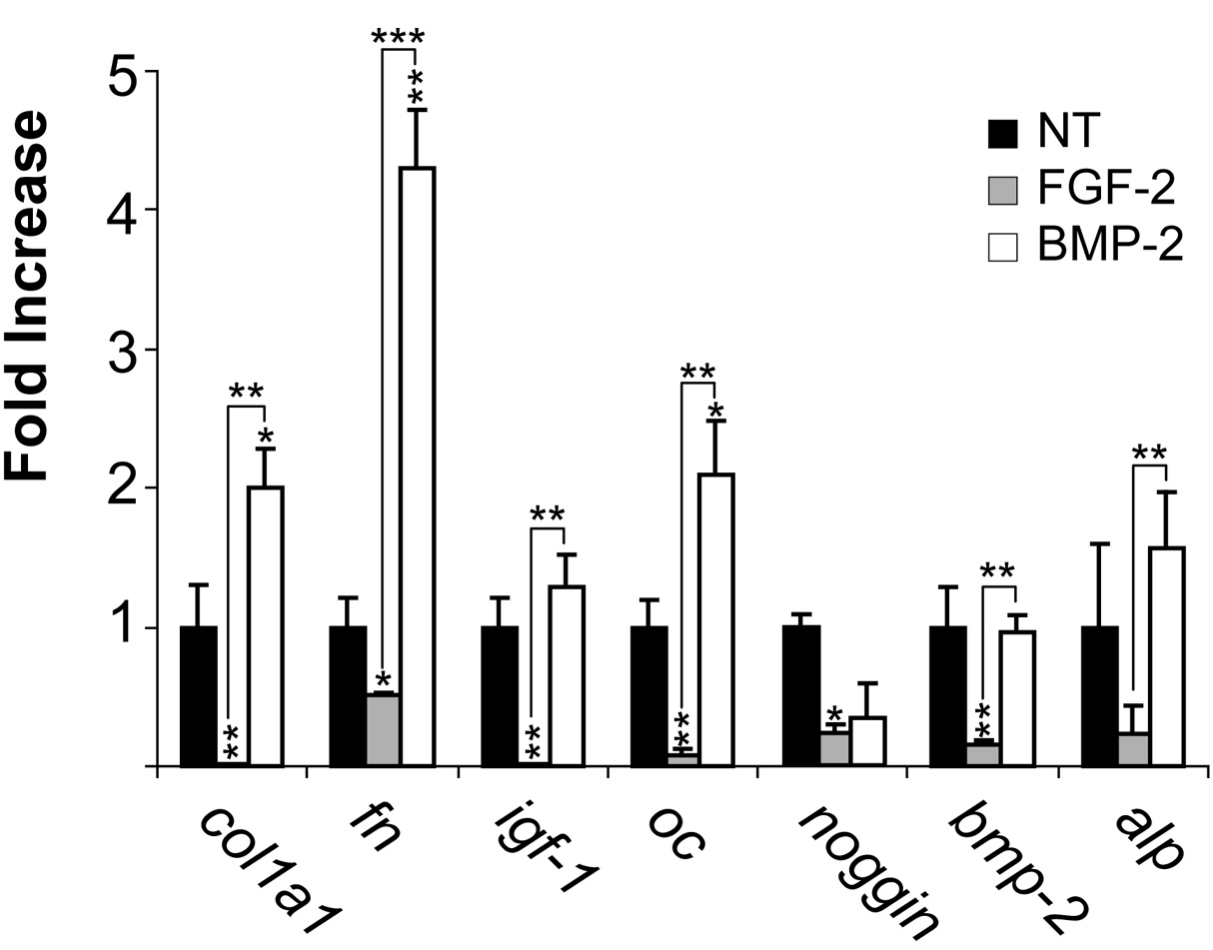
**FGF-2 and BMP-2, the yin and yang of mineralization: Contrast of effect of 24 hours of treatment with FGF-2 or BMP-2 on fold increase in abundance of mineralization-related gene expression**. Mineralizing MC3T3-E1 cells were prepared as described in Materials and Methods. They were then treated with either FGF-2 or BMP-2 for 24 hours at which time RNA was collected and analyzed for relative abundance using qRTPCR. Each bar represents mean ± SD triplicate independent biological samples each time point corrected to cyclophilin. (*p < 0.05; **p < 0.01 with two-tail student t-test compare to 0 hour of each gene.) *<0.05; **<0.01; ***<0.0001

Since FGF-2 increased growth associated genes, we used BMP-2, a known promoter of mineralization, to study relative abundance of gene expression in mineralizing cells after 24 hours of treatment. As seen in Table 2, we found that BMP-2 treatment caused significant increases in genes associated with mineralization including *cola1, fn, noggin *and *oc*. Moreover, BMP-2 treatment caused little or no changes in expression of genes associated with angiogenesis and migration e.g. VEGF and MMP3. When compared with relative gene abundance of FGF-2 treated cells (Figure 3) we found that in general, BMP-2 maintained the mineralizing RNA profile of *igf-1, alp*, and *bmp-2 *and significantly increased expression of other genes associated with mineralization like col1a1, *fn, ilgf-1, noggin *and *oc*. Fgf-2, on the other hand, significantly suppressed expression of mineralizing genes.

**Table 2:** Relative abundance of gene expression in FGF-2 and BMP-2 treated cells.

	Non-treated		FGF-2 treated		BMP-2 treated		FGF-2 vs BMP-2
Gene	Average	SD	Average	SD	Average	SD	p-value
**Collagen Type I**	**85,081.73**	2,5316.39	**678.21	358.27	*170,243.43	24,493.77	0.0003
**Fibronectin**	**55,827.93**	1,2119.18	*28,432.19	1195.92	**239,750.67	23,464.19	0.0001
**IGF1**	**3,249.41**	689.70	**50.65	13.30	**4,193.34**	739.19	0.0006
**RUNX2**	**349.09**	40.63	**674.95	63.04	**1,043.65**	783.29	n.s.
**VEGFA**	**109.49**	38.86	**5,132.66	755.22	**537.13**	379.66	0.0007
**TGFβ**	**93.08**	10.55	**245.40	41.93	*185.20	38.34	n.s.
**ALP**	**58.30**	34.81	**13.39**	11.68	**91.77**	23.15	0.0064
**OC**	**16.20**	3.19	**1.38	0.65	*34.04	6.11	0.0008
**Noggin**	**7.11**	2.77	*1.61	0.49	**2.41**	1.76	n.s.
**BMP-2**	**0.40**	0.12	**0.06	0.01	**0.38**	0.05	0.0004
**MMP3**	**0.03**	0.03	**4.04	0.97	**0.12**	0.14	0.0023

This table shows the relative abundance of gene expression in mineralizing MC3T3-E1 cells after 24 hours of treatment with FGF-2 (5 ng) or BMP-2 (100 ng). Total RNA was harvested 24 hours after the addition using Qiagen RNeasy kit. A two-step RT-qPCR was preformed. Each data point represents the mean ± SD of three biological independent samples. *p < 0.05; **p < 0.01; ***p < 0.0001 against 0 hour control samples with 2 tail student t test.

### Relative mineralization of FGF-2 and BMP-2 treated cells

**Figure 4:**
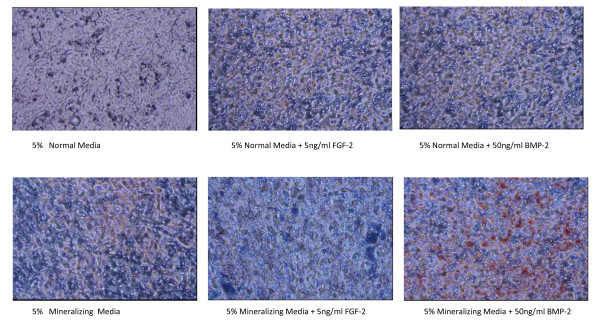
**Alizarin Staining of Mineralizing Osteoblast cells**. MC3T3-E1 osteoblasts were seeded at 3000 cells/well in 96 well CELLBIND^® ^plates in normal medium. Once cells were confluent, media was changed to 5% NM or 5% mineralizing media with or without 5 ng/ml FGF-2 or 50 ng/ml BMP-2. Two days after treatment, media was removed and cells were fixed in 10% formalin and stored at 4°C until subsequent analysis. Cells were stained for calcium with 2% Alizarin Red for 10 minutes and visualized under 20× objectives for photography. Many areas of mineralization, as seen by bright red staining, were present in the cells treated with 5% MM plus 50 ng/ml BMP-2 (FIG. 11). Little to no mineralization was seen with other 5 treatments.

As seen in Figure 4 and Table 3, BMP-2 treatment enhances mineralization of the cells as shown by uptake and presence of Alizarin Red after cultures were grown to confluence and then treated with BMP-2 or FGF-2 for 24 to 48 hours. Cells were then washed and stained with 2% Alizarin Red and results determined using photography or fluorescence analysis at 48 hours of treatment.

**Table 3:** Mineralization of cells with BMP-2

Treatment	Relative abundance
NM	5.6 ± 1.7
NM + 5 ng/ml FGF-2	5.3 ± .1
NM + 50 ng/ml BMP-2	16.2 ± 4.2
MM	9.1 ± 2.0
MM + 5 ng/ml FGF-2	4.9 ± 1.1
MM + 50 ng/ml BMP-2	55.2 ± 12.7

The Alizarin Red (2%) stained cells were incubated with 10% acetic acid for 30 minutes to release bound Alizarin Red into solution. The solution was neutralized with 10% ammonium hydroxide and the absorbance of Alizarin Red was measured at 450 nm using a microplate reader (n = 6). Data is expressed at in absolute amounts according to a standard curve.

## Discussion

Bone formation during injury repair is a multi-step series of events modulated by an integrated cascade of gene expression that initially supports the proliferation stage. The later mineralization stage is associated with the sequential expression of genes that support biosynthesis, organization and mineralization of the bone extracellular matrix. Mineralization requires expression of structural proteins such as collagen type I, osteocalcin, as well as noggin and runx2 which aid in mineralization [1]. Transcriptional control defines the regulatory events necessary for both stages of bone formation [23]. There is a general consensus that during injury GFs are released from the wounded bone matrix and promote healing [24]. In this study, we have documented the relative efficiency of bone growth factors FGF-2, TGFβ, and PGE2 markedly enhanced the synthesis of the total protein content of the dishes (Table 1)

Rate of proliferation was dependent on the specific GF. FGF-2, TGFβ and PGE_2 _significantly promote growth, with FGF-2 having the highest efficacy and the lowest dose. FGF-2 produced a distinct pattern of gene expression. FGF-2 down regulates genes associated with mineralization while it induces genes associated with proliferation and angiogenesis, a finding supported by observations of others [25]. Since cox-2 had a 27-fold induction by FGF-2, we examined the effect of the COX-2 product, PGE_2 _on proliferation. We found that PGE_2 _increased DNA synthesis by 3.3 fold significantly higher than TGFβ, IGF-1, PDGF, suggesting that its induction by FGF-2 helps complete the FGF-2 full induction of osteoblast growth. These data also suggest that FGF-2 may be an important regulator of migration, angiogenesis and proliferation during the first stage of healing a critical defect since it induces *mmp3, vegfa *and *vegfr1 expression*. In data not shown, FGF-2 had no effect on expression of mmp-1. Moreover, FGF-2 induced its own message as well as TGFβ, but significantly reduced expression of BMP-2, osteocalcin, noggin, runx2, collagen type I and IGF-1, genes which are associated with mineralization.

As described by others, bone formation is divided into two phases, proliferation and mineralization [2,26-29]. These two stages are the result of a specific sequential regulation of gene expression from the early phase of osteoblast proliferation to the final steps of mineralization. Once the cells start mineralizing, cell division and DNA synthesis dramatically slow down and eventually cease. When an injury occurs in mineralized tissue, GFs like FGF-2 are released and start a new proliferation stage to heal the defect. The increase in cell replication in a mineralizing cell likely represents a de-differentiation from the mineralizing phase to the growing phase, and increases expression of GFs most likely induce proliferation. Treatment of the mineralized defect model with FGF-2 resulted in gene expression that corresponds to de-differentiation (e.g. significant increases in growth related genes *egf-1, fgf-2*, *cox-2, TGFβ, vegfA, vegfr *and *mmp3 *and down-regulation of mineralizing related genes). Vegf and vegfr1 are primary regulators of angiogenesis, while MMP3 is thought to play a major role on cell behaviors such as proliferation and migration [30] which may explain the ability of the FGF-2 to enable the cultured cells to fill the defect void efficiently. The fact that FGF-2 induces its own expression suggests that after injury, the FGF-2 released from the wound matrix could promote it's own expression, making it a feed-forward loop.

Although Figures 1 and 2 demonstrate the relative FGF-2 regulation and sequential expression of growth, angiogenic and chemotactic genes and depresses expression of mineralization-related genes, these figures do not tell us the *relative abundance *of the genes. In Table 2, we determined the relative abundance of genes in three groups after 24 hours; with or without treatment with FGF-2 or BMP-2. FGF-2 caused a significant increase in abundance of genes associated with proliferation, chemotaxis and angiogenesis. Moreover, the addition of FGF-2 to the mineralized wounded cultures caused a marked decrease in abundance of *col1a1 *as well as *fn, igf-1, noggin, oc, bmp-2 *and *alp *message. In the early stages of mineralization, the major protein (greater than 20%) synthesized by the osteoblast is collagen, however collagen is not a major component of the proliferating cell, suggesting that FGF-2 stimulates proliferation partly through its ability to drastically reduce the relative abundance of a majority of the mineralizing-associated genes. The dramatic reduction of IGF-1 by FGF-2 suggests a role for IGF-1 in mineralization, this is in agreement with findings of others that demonstrated IGF-1 to be a major factor in bone mineralization [31-33] using the IGF-1 null mouse. In contrast, in cells treated with BMP-2, the relative abundance of *col1a1, fn*, *oc*, and *tgfβ *were dramatically induced while BMP-2 had no significant effect on genes related to growth, angiogenesis or chemotaxis. These data suggest that BMP-2 may be the best GF to use for the mineralization stage but not the proliferation stage of bone formation. This finding may help explain studies by others [34] who discovered that a *delayed *administration of BMP-2 to a fracture resulted in better repair of critical size defects. It is likely that the delay of BMP-2 treatment allowed a longer period of proliferation prior to BMP-2 promotion of mineralization. Our findings in Table 2, 3 and Figure 3 support the hypothesis that FGF-2 and BMP-2 are required at different stages of bone repair.

## Conclusions

These data demonstrate the de-differentiation (reduction of mineralization genes) effect of FGF-2 likely plays a key role in osteoblast proliferation, the first stage of bone formation. Some have expressed concern that *ex-vivo *proliferation of human stem cells by a growth factor like FGF-2 might change the osteogenic characteristics of a pre-osteoblast; however others have shown that expansion of the population does not affect later osteogenic potential [35] of stem cells. Therefore, an expansion of osteoblast cells by FGF-2 might be an excellent strategy for first stage re-population of a critical defect since FGF-2 has the needed efficacy for promoting proliferation. These data also suggest that the final stage of bone repair is best accomplished with BMP-2 due to its promotion of differentiation and mineralization.

## Competing interests

The Department of Veterans Affairs has filed and owns a patent using some of the data found in this manuscript.

## Authors' contributions

MHF conceived the study, designed the study, directed the research and wrote the manuscript. C-FL made substantive intellectual contribution in the acquisition of data, analysis and has contributed to the manuscript. Both authors have read and approved the final manuscript.
